# Dermatomyositis Related Paraneoplastic Encephalitis Presenting as Terminal Delirium in the Palliative Care Unit – A Case Report

**DOI:** 10.1177/08258597221074425

**Published:** 2022-01-24

**Authors:** Kelvin Lou, Shikha Minhas, Shalini Nayar

**Affiliations:** 1 University of British Columbia, British Columbia, Canada; 2 University of British Columbia, British Columbia, Canada

## Abstract

Advanced cancers can have many complications including paraneoplastic processes such as dermatomyositis, hypercalcemia and encephalitis. Due to the wide-ranging nature of symptoms, the diagnosis requires a high index of suspicion and often requires a clinical diagnosis while awaiting for laboratory confirmation. Managing paraneoplastic phenomenon at the end of life requires robust goals of care conversations to determine what the patient is willing to go through to achieve specific goals. We present the case of a 50-year-old male with a history of squamous cell carcinoma presenting as unresolving delirium secondary to paraneoplastic encephalitis.

## Introduction

Paraneoplastic phenomenon are rare but well described cancer related complications.^1^ Due to the wide- ranging presentations, the diagnosis of paraneoplastic processes can be difficult to make. Patients may also struggle against the stigma that comes with having a terminal illness, where investigations and treatments may be withheld without a thorough discussion regarding goals of care.^2^ Within the palliative care setting, the diagnostic process is further complicated by the patient's limited prognosis, fluctuating ability to consent and the need to limit over-investigation. The tension between palliation and treating reversible causes forms a complex interplay near the end of life. Within the multitude of cancer complications, there needs to be a patient centered approach to determine the individualized goals and the acceptable tradeoffs. Paraneoplastic phenomenon represent a unique case of this kind of delicate balance, where the diagnosis and empiric treatments are made within a setting of clinical uncertainty. Explaining the diagnostic uncertainty to the patient in an emotionally charged situation plays a key role in goals of care discussions and informed decision-making.

## Case Presentation

We present the case of a 50-year-old male with a history of late presenting squamous cell carcinoma. At time of his oncological diagnosis he was found to have significant tumor burden and bony metastasis. He was subsequently lost to follow up and was later admitted to the hospitalist service with confusion and generalized weakness. Collateral history suggests that the patient likely has had worsening confusion and weakness over 3–4 weeks associated with a rapid functional decline from a previously robust state. At the time of admission by the hospitalist service, the neurological examination was documented as grossly intact and there was no documentation of any abnormal dermatological findings. Laboratory investigation on admission show an ionized calcium of 2 mmol/L (N 1.05-1.3 mmol/L), an elevated WBC of 20 × 109/L (N 4.0-10 × 109/L) and CRP 36.5 mg/L (N 0.0-8.0 mg/L). The infectious workup including urine and blood cultures were negative. Review of the laboratory work shows recurrent hypercalcemia despite repeated treatment with crystalloid fluid.

After one week of being bedbound and with no improvement to his cognition despite repeated correction of his hypercalcemia, he was transferred to the palliative care inpatient unit for end of life care in the context of terminal delirium. Upon arrive to the palliative care unit, a neurological examination revealed proximal muscle weakness with no focal deficits and a supple. Dermatological examination revealed a scaly red rash along the metacarpal-phalangeal (MCP) joints bilaterally consistent with Gottron's papules. There was no heliotrope rash and examination of the other joints was unremarkable. Despite review of the chart and discussion with the nursing staff, we are unable to confirm whether this rash was present at admission or developed in hospital. Additional workup for causes of muscle weakness and autoimmune disease at this time revealed vitamin B12 463 pmol/L (N 148-590 pmol/L), vitamin D 1,25-Dihydroxyvitamin D 97 pmol/L (N 60-156 pmol/L), 25-Hydroxyvitamin D 20 nmol/L (N 62-200 nmol/L), thyroid stimulating hormone (TSH) 1.6 mU/L (N 0.5-5.0 mU/L), negative antinuclear antibody (ANA) and antineutrophil cytoplasmic antibody (ANCA). Of note is that the creatine kinase (CK) was within normal range at 42 U/L (N 30-170 units/L) throughout the entire hospital. A head MRI showed small white matter changes but no sign of intracranial metastasis or leptomeningeal involvement. MRI of the proximal muscles of the thighs showed findings suggestive of inflammatory myositis.

Given the constellation of symptoms of proximal muscle weakness and Gottron's papules in the setting of a lung malignancy, a provisional diagnosis was made of dermatomyositis. In regards to the unresolving delirium, given that other causes such as metabolic, drug or infection had been ruled out, consideration was given for rarer causes such as paraneoplastic phenomenon. In discussion with radiology, the head MRI white matter changes could possibly be consistent with paraneoplastic encephalitis. In an effort confirm a paraneoplastic process, additional workup was done including a lumbar puncture and muscle biopsy. The samples were sent to an outside laboratory for the paraneoplastic and myositis laboratory panels respectively.

An oncology opinion was sought but the patient was deemed too weak to tolerate chemotherapy. Understanding that his prognosis was short, the main goal expressed by the patient was to see his wife and child to say farewell. The challenge was that his wife and child were living overseas and have been unable visit him due to travel restrictions. His fluctuating delirium also made it challenging to hold a coherent conversation. While awaiting these results, the patient's mental status continued to worsen and the shared decision was made to trial empiric pulse steroid therapy with methylprednisolone 1g/day for three days. Over the course of three days of treatment, there was marked improvement to his cognition, to the point where he was able to unlock and operate his cellphone and hold a coherent conversation with his wife. His clinical status then deteriorated into a terminal delirium despite continued steroid therapy and he passed away shortly thereafter. After the patient's passing, the pending investigations returned showing positive Anti-NXP2, confirming a diagnosis of dermatomyositis. The MRI findings of non-specific white mater changes were re-discussed with the radiologist with the added clinical history and it was determined that it could be compatible with either a diagnosis of CNS vasculitis or a paraneoplastic encephalitis. The results of the paraneoplastic panel was negative, with the caveat that only a limited number of antibodies are tested at the particular laboratory.

## Discussion

Dermatomyositis is an inflammatory myopathy with a known association with malignancies and can present as part of a constellation of paraneoplastic processes.^1^ Typical signs and symptoms include proximal muscle weakness associated with characteristic rashes such as heliotrope rashes, shawl sign and Gottron's papules.^1^ Typical laboratory abnormalities include elevated CRP and CK but in the palliative setting these findings may present atypically. In the presented case, a diagnostic cofounder was that the cancer related cachexia may have resulted in a lower CK at baseline.^3^ In regards to the etiology of the terminal delirium, there are rare case reports of dermatomyositis related vasculitis but given the marked improvement on steroids, a paraneoplastic encephalitis is also possible. Paraneoplastic phenomenon include a diverse range of conditions that can have wide ranging systemic effects. For suspected paraneoplastic neurological disorders, the standard investigation is testing for autoantibodies directed against neural antigens.^4^ Due to the rare nature of these conditions, there are only some that can be tested for, with many other autoantibodies still under investigation.^4^ The further difficulty is that there are no established diagnostic criteria for many of these conditions and the often would require an empiric clinical diagnosis while awaiting for laboratory confirmation. Treatments for paraneoplastic encephalitis include intravenous immunoglobulin (IVIG) and high dose steroids.^4^ In the presented case, another clue that a paraneoplastic process was active was the recurrent hypercalcemia, a known phenomenon driven by PTH-related peptide (PTH-rP),^5^ which unfortunately was not available at the testing laboratory. The ongoing diagnostic challenge of palliative medicine is to differentiate the complex overlap of end-of-life symptoms versus potentially reversible causes. In the presented case, the generalized fatigue and rapid functional decline likely masked the proximal muscle weakness and delayed the diagnosis of dermatomysitis. Once a single paraneoplastic process was identified, the constellation of symptoms led to the unifying diagnosis of a paraneoplastic process as the cause of the terminal delirium. This diagnosis further opened up treatment options for a steroid responsive cause of delirium. The diagnosis of paraneoplastic phenomenon is challenging at the best of times and is even more so in a palliative setting. In the presented case, the symptoms were suggestive of a paraneoplastic progress but the confirmatory tests took weeks to return and was unhelpful for making medical decisions in the moment. Explaining diagnostic uncertainty in an emotionally charged situation plays a key role in goals of care discussions. Part of that conversation is determining what is the acceptable level of diagnostic uncertainty to warrant empiric treatment. The tension between palliation and treating reversible causes is resolved through robust goals of care conversations to determine what the patient is willing to go through to achieve specific goals. In the presented case, the goal was to regain temporary cognitive capacity to say farewell to his wife in the midst of terminal delirium. Based on this goal, the patient was willing to trial an empiric course of steroids without laboratory confirmation. The presented case lies within a narrow space where the prognosis is limited but small precious movements can still be regained. What constitutes reasonable medical care in these situations needs to be individualized to the patient and each clinician has to answer that question for themselves. Patient centered care within palliative care requires ongoing advocacy against the stigma carried by patients with a terminal diagnosis. Although some may see temporarily reversing a delirium in a dying patient as futile care, we would strongly argue that end-of life is an even more critical time to explore and respect a patient's goals of care.
